# Revolutionizing Radiology With Artificial Intelligence

**DOI:** 10.7759/cureus.72646

**Published:** 2024-10-29

**Authors:** Abhiyan Bhandari

**Affiliations:** 1 General Medicine, Wexham Park Hospital, Slough, GBR

**Keywords:** artificial intelligence, diagnosis, patient care, radiology, workflow optimization

## Abstract

Artificial intelligence (AI) is rapidly transforming the field of radiology, offering significant advancements in diagnostic accuracy, workflow efficiency, and patient care. This article explores AI's impact on various subfields of radiology, emphasizing its potential to improve clinical practices and enhance patient outcomes. AI-driven technologies such as machine learning, deep learning, and natural language processing (NLP) are playing a pivotal role in automating routine tasks, aiding in early disease detection, and supporting clinical decision-making, allowing radiologists to focus on more complex diagnostic challenges.

Key applications of AI in radiology include improving image analysis through computer-aided diagnosis (CAD) systems, which enhance the detection of abnormalities in imaging, such as tumors. AI tools have demonstrated high accuracy in analyzing medical images, integrating data from multiple imaging modalities such as CT, MRI, and PET to provide comprehensive diagnostic insights. These advancements facilitate personalized treatment planning and complement radiologists' workflows.

However, for AI to be fully integrated into radiology workflows, several challenges must be addressed, including ensuring transparency in how AI algorithms work, protecting patient data, and avoiding biases that could affect diverse populations. Developing explainable AI systems that can clearly show how decisions are made is crucial, as is ensuring AI tools can seamlessly fit into existing radiology systems. Collaboration between radiologists, AI developers, and policymakers, alongside strong ethical guidelines and regulatory oversight, will be key to ensuring AI is implemented safely and effectively in clinical practice.

Overall, AI holds tremendous promise in revolutionizing radiology. Through its ability to automate complex tasks, enhance diagnostic capabilities, and streamline workflows, AI has the potential to significantly improve the quality and efficiency of radiology practices. Continued research, development, and collaboration will be crucial in unlocking AI's full potential and addressing the challenges that accompany its adoption.

## Introduction and background

Artificial intelligence (AI) is a branch of computer science focused on creating algorithms and systems that are capable of emulating human intelligence. These systems are designed to understand and reason by processing vast datasets to make autonomous decisions. AI has revolutionized industries such as business, education, finance, and healthcare, bringing transformative advancements that streamline operations and improve outcomes [1]. In healthcare, applications of AI include AI-assisted surgical robots and algorithms that aid in early disease detection [2]. In radiology, key AI subspecialties include machine learning for analyzing complex patterns across imaging modalities, deep learning for enhancing image interpretation and workflow optimization, and natural language processing (NLP) to assist with report writing and clinical decision-making [3-5].

The term "artificial intelligence" was first introduced by John McCarthy during the 1956 Dartmouth Conference, marking the formal birth of the field [6]. Early AI applications in fields such as machine learning and robotics were made possible by advances in data science and computing power [7]. AI in radiology has made significant strides since its early beginnings. One of the first major implementations occurred in the 1980s with the introduction of computer-aided detection (CAD) software in mammography, which played a key role in improving the accuracy of lesion detection [8]. Since then, AI's integration into radiology has advanced substantially, which has resulted in enhanced diagnostic accuracy in various radiology subspecialties and optimized workflows [9,10].

Advanced deep learning algorithms have demonstrated remarkable performance in medical image analysis, contributing to early disease detection and personalized treatment approaches [11,12]. These tools support radiologists by streamlining tasks such as image interpretation, anomaly detection, and clinical decision-making, ultimately reducing the time required for routine processes and allowing radiologists to focus on more complex cases [13,14].

Tumor detection serves as a prime example of AI's potential in radiology. AI-based tools have shown great promise in automating lesion detection, characterization, and segmentation, aiding in early diagnosis and improving patient outcomes. For example, AI algorithms can match or even surpass the accuracy of experienced radiologists when distinguishing between benign and malignant lung nodules [15,16]. Various techniques, such as deep learning and convolutional neural networks (CNNs), are being explored and refined for lung cancer imaging analysis [17].

AI is also reshaping radiology workflows, enhancing both efficiency and accuracy at multiple stages, including imaging requests, scan scheduling, and image interpretation [18]. By automating routine and time-consuming tasks, AI enhances workflow efficiency, allowing radiologists to focus on more complex cases [14].

## Review

Applications of AI in various sectors of radiology

Artificial intelligence (AI) has brought transformative advancements to radiology, enhancing diagnostic capabilities across several subfields. These AI-driven technologies help radiologists by improving accuracy, speed, and consistency in medical image interpretation, leading to better patient outcomes. Below are some specific applications of AI in various subfields of radiology.

Neuroradiology

In the realm of neuroradiology, AI is widely used for detecting brain tumors, assessing neurodegenerative diseases, and diagnosing conditions such as intracranial hemorrhage and strokes. AI-based systems such as Aidoc have proven effective in diagnosing intracranial hemorrhage, offering automated tools that assist radiologists in early detection [19]. This enhances diagnostic efficiency, particularly in high-pressure environments such as the emergency department.

AI has significantly enhanced the accuracy of brain tumor detection, particularly gliomas. In a recent study, AI algorithms using deep learning demonstrated an area under the curve (AUC) of 93.2% in distinguishing between low-grade gliomas and high-grade gliomas using MRI data. These results suggest that AI can outperform traditional diagnostic methods in assessing tumor grade, facilitating early and precise treatment decisions [20]. Advanced imaging techniques such as functional MRI, ultra-high-field MRI, diffusion tensor imaging (DTI), PET/CT, and deuterium magnetic resonance spectroscopy (2H MRS) further enhance the precision of AI-driven approaches. These technologies are critical in surgical planning, helping to target tumors more accurately and improve outcomes by minimizing damage to surrounding tissue and reducing complications [21].

Moreover, deep learning algorithms have been employed to identify critical findings such as hemorrhage, mass effect, and hydrocephalus in CT head scans. These algorithms are being integrated into automated screening systems, which could alert clinicians to life-threatening conditions in real time, streamlining workflows and improving response times [22].

Breast Imaging

AI systems have shown remarkable capabilities in recognizing subtle differences in breast tissue that may be imperceptible to the human eye, identifying tumors with precision comparable to experienced radiologists. AI, particularly deep learning, has significantly enhanced the accuracy and effectiveness of mammography, a key tool in breast cancer detection [23]. In a study involving 22,621 mammograms, the AI software achieved an AUC of 89.6% in detecting breast cancer. This performance illustrates AI's potential to enhance early detection, particularly in distinguishing interval cancers that are often missed in traditional screenings [24]. This advanced detection capability has been instrumental in the early identification of breast cancer, which is particularly critical as the incidence of this disease has increased, especially among younger populations.

Data from digital mammography and digital breast tomosynthesis are being leveraged by AI algorithms to target several critical aspects of breast cancer risk assessment. These include providing robust and reproducible measurements of breast density, which is a well-established risk factor for breast cancer, assessing a patient's risk of developing breast cancer, and identifying patients who are likely to develop aggressive or rapidly growing tumors, even after a negative or routine screening, often due to masking effects [25]. Additionally, AI is aiding in predicting tumor progression and aggressiveness, enabling earlier, more personalized interventions that improve patient outcomes by guiding treatment decisions based on prognosis [26].

Chest Radiology

AI-based systems have been developed to identify a range of chest pathologies, including pneumonia, pulmonary nodules, tuberculosis, and interstitial lung diseases [27]. These AI tools have shown promising accuracy in detecting thoracic abnormalities [28]. For example, in a study using the CheXNet deep learning algorithm, AI achieved an AUC of 86.2% for atelectasis detection, performing better than radiologists [29]. AI-based systems have also demonstrated high accuracy in analyzing chest radiographs for early pneumonia diagnosis, offering a powerful adjunct to traditional radiological assessments [30].

AI tools are also being increasingly utilized in emergency settings to assist radiologists in detecting and evaluating common chest pathologies, such as pneumothorax, pneumonia, heart failure, and pleural effusion, on chest X-rays. These technologies enhance the speed and accuracy of diagnoses, particularly in critical care environments, where rapid decision-making is crucial [31].

Cardiovascular Radiology

AI techniques, including machine learning and deep learning, are making significant contributions to cardiovascular radiology, particularly in coronary artery disease detection, cardiac function assessment, and aortic aneurysm detection. These techniques are being applied across various imaging modalities such as CT, MRI, and nuclear perfusion imaging, automating tasks such as calcium scoring, quantifying coronary stenosis, plaque analysis, and myocardial tissue characterization. This automation enhances diagnostic precision and efficiency, allowing radiologists to focus on complex cases [32].

In echocardiography, AI is used to automate and standardize image analysis, improving the assessment of cardiac function. AI-based algorithms streamline the measurement of cardiac volumes, ejection fractions, and wall motion abnormalities, reducing variability and improving diagnostic consistency [33]. This leads to more accurate and reproducible assessments.

AI is playing an important role in the detection and management of aortic aneurysms by improving image segmentation and automating measurements. This allows radiologists to more precisely characterize aneurysm morphology. Furthermore, AI facilitates the analysis of large datasets, enabling the identification of patterns that predict aneurysm growth and rupture risk. By providing these insights, AI helps clinicians determine the likelihood of needing surgical intervention, allowing for more timely and personalized treatment planning and ultimately improving patient outcomes [34].

Abdominal Radiology

In abdominal radiology, AI plays a significant role in organ segmentation, lesion detection, classification, and prognosis prediction. For example, deep learning has been applied to liver imaging, where it assists in the detection and evaluation of focal liver lesions. These AI systems help radiologists differentiate between benign and malignant liver lesions, improving diagnostic accuracy and aiding in treatment planning [35,36].

AI-driven approaches are also transforming pancreatic cancer detection, addressing the challenges of late detection and poor prognosis that are common with this disease. AI models can analyze imaging data to identify early signs of pancreatic cancer, potentially allowing for earlier intervention and improved patient outcomes. These approaches aim to overcome the current limitations in diagnosing this aggressive cancer, where early detection is often difficult [37].

Furthermore, AI has shown promise in the detection and management of renal calculi as well. Algorithms applied to CT and ultrasound imaging demonstrate high accuracy in identifying renal calculi. Machine learning models are also being used to predict stone composition, recurrence rates, and outcomes for treatments such as percutaneous nephrolithotomy and extracorporeal shockwave lithotripsy. These tools assist in personalized treatment planning, ensuring better outcomes for patients [38,39].

Musculoskeletal Radiology

Artificial intelligence (AI) is transforming musculoskeletal radiology by enhancing the accuracy and efficiency of diagnostic tasks. AI systems are particularly useful for detecting fractures, identifying joint abnormalities, assessing bone diseases, and performing bone age assessments. In addition, AI algorithms are employed to monitor the progression of arthritis, assisting in both diagnosis and long-term management [40].

Deep learning models have demonstrated high accuracy in fracture detection across various imaging modalities, such as X-rays, CT scans, and MRI. These models have shown exceptional performance in detecting specific fracture types, such as hip fractures [41]. AI's ability to quickly and reliably identify fractures not only aids radiologists in making faster diagnoses but also improves overall patient outcomes by enabling more timely interventions.

AI is also increasingly used in the management of rheumatoid arthritis (RA), where it offers promising improvements in screening, diagnosis, and monitoring. Machine learning and deep learning techniques analyze a variety of data sources, such as electronic health records (EHRs) and imaging data, to assist in the early detection of RA, monitor disease progression, and assess treatment responses. This technology enables personalized treatment plans, improving long-term management of the disease [42,43].

Nuclear Medicine

AI applications in nuclear medicine have progressed significantly, with key roles in image generation, reconstruction, and quality enhancement for modalities such as PET and single photon emission computed tomography (SPECT). These technologies help reduce acquisition time and improve image quality through AI-based denoising and deblurring techniques [44]. AI has also been pivotal in areas such as automated planning, dosimetry, and procedure execution, improving the precision and reliability of nuclear medicine interventions. In particular, AI has shown strong potential in tasks such as dose optimization, image corrections, and reconstruction, where AI-driven models enhance diagnostic capabilities [45].

Recent developments highlight AI's ability to address key challenges in nuclear medicine, such as improving the quality of PET and SPECT imaging. Advanced deep learning techniques, such as convolutional neural networks (CNNs), have outperformed traditional methods in reducing noise and enhancing resolution in PET scans, leading to more accurate diagnostics, especially for conditions such as cancer and cardiac diseases [46,47]. AI is also increasingly applied to automate image reconstruction processes, facilitating faster and more accurate interpretations. AI-driven methods are being actively integrated into clinical workflows to optimize the use of these imaging modalities and improve patient outcomes [48].

Interventional Radiology (IR)

In interventional radiology (IR), AI is playing a growing role in decision support, outcome prediction, and imaging technique improvement [49]. AI technologies assist in lesion detection, segmentation, and interpretation, which enhances procedural accuracy. AI's potential is particularly evident in interventional oncology, where it provides prognostic insights, assists in identifying the optimal catheter or ablation points, and improves real-time image interpretation during procedures [50].

Moreover, AI systems are proving beneficial for pre-procedural planning and real-time navigation. For example, machine learning algorithms are being used to predict patient-specific outcomes for procedures such as radiofrequency ablation and transarterial chemoembolization, offering more precise and personalized treatment strategies [51]. These AI advancements hold great promise in transforming how radiologists conduct complex IR procedures, increasing safety and efficiency.

Improving patient care with AI

AI is transforming patient care in radiology by enhancing decision-making, improving workflow efficiency, reducing radiation exposure, and facilitating personalized treatment approaches. This section delves into key areas where AI is directly improving patient care in radiology.

Decision Support for Imaging Requests

AI has proven invaluable in supporting clinical decision-making, particularly in determining whether an imaging request is appropriate based on a patient's medical history. AI can assist in choosing the most suitable imaging modality, providing guidance aligned with the American College of Radiology (ACR) appropriateness criteria, which ensures evidence-based recommendations for imaging and treatment decisions. By incorporating these guidelines, healthcare providers can enhance the quality of care while optimizing the use of radiological resources. AI-powered chatbots are also being employed to advise referring clinicians, helping them select the most appropriate imaging studies based on clinical needs [52].

Using AI for Scheduling Scans

AI-powered triage systems are transforming the scheduling of imaging scans by prioritizing scans based on clinical urgency. This ensures that critical cases are attended to promptly, ultimately improving patient outcomes. In addition to managing waiting times and prioritization, AI-based systems provide opportunities to refine service offerings, leading to improved patient satisfaction and more efficient workflow management [18].

Reducing Radiation Exposure

AI is playing a crucial role in minimizing patient exposure to radiation, particularly in modalities such as PET scanning. Deep learning models are used to reconstruct high-quality images from low-dose PET scans, thereby reducing the overall radiation exposure while maintaining diagnostic accuracy [53]. This has important implications for cancer screening and staging programs, where concerns about high radiation doses often deter the use of PET. AI-enabled fluoroscopy systems also contribute by significantly reducing radiation exposure to both patients and healthcare personnel through the use of ultrafast collimation techniques that focus radiation on the region of interest [54]. Moreover, AI techniques, such as deep learning for image reconstruction and optimization of acquisition parameters, have reduced radiation doses in CT scans by improving image quality and reducing noise [55].

Faster Scans

AI has the potential to reduce the time required for imaging scans, including CT and PET scans, by optimizing image acquisition protocols and improving image reconstruction techniques. This reduces patient exposure to radiation and minimizes the time spent on the procedure itself [56]. Machine learning algorithms are now capable of automating tasks such as image normalization, quality improvement, noise reduction, and segmentation, which streamline the entire imaging process [57,58].

Additionally, AI can dynamically adjust scanning protocols before or during the procedure, depending on patient-specific factors and early imaging sequences, optimizing sequence selection and decreasing scan times. This is particularly beneficial for MRI, which traditionally involves long acquisition times, improving patient comfort and increasing throughput [59].

Improving the Quality of Images

AI has shown great promise in enhancing image quality, a crucial aspect of diagnostic radiology. Advanced algorithms, especially those based on deep learning, can automatically process medical images to reduce noise and artifacts, improve spatial resolution, and enhance visualization of anatomical structures and lesions. These improvements lead to more accurate diagnoses and better patient outcomes [57]. Machine learning also contributes to better image quality by reducing image noise, compensating for motion artifacts, and improving spatial resolution [59].

Personalized Medicine in Oncology

AI is revolutionizing personalized care in oncology, particularly in radiation therapy and precision medicine. AI aids in creating optimized treatment plans by segmenting organs, tissues, and tumors more accurately, thereby improving targeting precision and reducing damage to surrounding healthy tissue. These advancements are vital for both radiation therapy and surgical planning, where accurate tumor segmentation is essential. Additionally, AI plays a key role in analyzing patients' genetic and molecular profiles, providing personalized treatment approaches in oncology. This facilitates tailored therapies that are more effective and reduce the risk of adverse side effects [60].

Enhancing workflow efficiency with AI

AI is streamlining radiology workflows by automating routine tasks and improving diagnostic efficiency. From flagging abnormal findings to speeding up report generation and image interpretation, AI helps radiologists work more efficiently while focusing on complex cases. The following sections highlight the key ways in which AI enhances workflow productivity in radiology.

Flag Abnormal Findings

AI has the ability to automatically flag abnormal or concerning findings in radiological images, a feature that can be integrated into picture archiving and communication systems (PACS). By flagging critical or suspicious findings, AI can help radiologists prioritize urgent cases, enabling more timely diagnoses and prompt treatments [61]. This system enhances diagnostic efficiency by ensuring that scans requiring immediate attention are reviewed first, streamlining the workflow in high-volume radiology departments.

More Efficient Reporting Workflows

AI has the potential to significantly improve the efficiency of reporting workflows. One key advancement is the use of automated hanging protocols, where AI pulls up relevant prior imaging studies and patient data from electronic health records (EHRs). This integration provides radiologists with crucial context at the point of diagnosis, reducing the time spent manually searching for prior scans and relevant clinical information [62]. AI integration into EHR systems also aids in reducing variability in follow-up recommendations, thereby improving the overall quality of reports.

Furthermore, AI systems can pre-populate reports with AI-generated findings, expediting the reporting process and improving consistency. AI can identify structures within an image and flag those for closer review or even load those images first, minimizing the time from scan loading to interpretation. This structured reporting pipeline significantly reduces manual effort, ensuring more comprehensive and faster reporting compared to traditional methods [63].

Automated Image Interpretation

AI tools also assist in image interpretation through the automation of complex tasks such as lesion measurements and volumetric calculations. Tools based on the Response Evaluation Criteria in Solid Tumors (RECIST) framework, which is the gold standard for assessing tumor response to cancer treatments, are labor-intensive when performed manually. AI-powered auto-segmentation tools can quickly and accurately calculate tumor sizes and volumes, drastically reducing the time radiologists spend on these tasks while improving consistency and accuracy [64].

AI further enhances the reporting process by incorporating natural language processing (NLP). NLP tools can generate preliminary reports based on detected image findings, saving radiologists time in creating reports from scratch. For instance, NLP-driven algorithms can organize unstructured dictation into coherent reports, optimizing the reporting process and ensuring more efficient and standardized documentation [5].

Challenges and limitations of AI in radiology

While AI holds great promise for transforming radiology, several challenges and limitations must be addressed to fully harness its potential. Understanding and overcoming these obstacles is essential for the safe, effective, and equitable integration of AI into radiology practice.

Data Quality and Quantity

One of the primary challenges in implementing AI in radiology is the need for large, high-quality datasets to effectively train algorithms. Medical data must be diverse and well-annotated, which is difficult to obtain due to patient privacy concerns, as well as the time-consuming nature of data labeling. Federated learning offers a promising solution, allowing algorithms to be trained across decentralized devices without sharing raw data. This technique preserves privacy while enabling healthcare providers to collaboratively enhance AI models across institutions.

In addition, radiologists and clinicians may lack training in handling big data, further complicating AI implementation. Big data requires specialized methods for handling its volume, variety, and velocity, which can pose an additional challenge in clinical environments where AI models need to process large-scale and heterogeneous datasets [65].

Integration and Interoperability

For AI to be successfully integrated into radiology, it must work seamlessly with existing infrastructure, such as radiology information systems (RIS) and picture archiving and communication systems (PACS). Achieving this level of interoperability across different vendors and healthcare institutions is a significant challenge, often requiring standardization of platforms and protocols. Without robust interoperability, AI adoption in radiology could face obstacles, especially when scaling across multiple institutions. Poor integration of AI tools not only limits their effectiveness but can also disrupt clinical workflows and create inefficiencies. Therefore, AI systems must be designed to complement existing radiology processes, enhancing workflow efficiency and supporting radiologists and clinicians rather than introducing new challenges [66,67].

Algorithm Transparency

As AI becomes more prevalent in radiology, several ethical and regulatory challenges need to be addressed. One of the most significant concerns is algorithm transparency. Many AI models, particularly deep learning systems, function as "black boxes," making it difficult to understand how they arrive at certain conclusions. This lack of transparency can pose challenges in clinical decision-making, as clinicians need to trust and understand AI-driven diagnoses before applying them in patient care. Explainable AI (XAI) is an emerging field that aims to make AI models more interpretable by providing insights into the features influencing AI decisions, helping to build confidence in the reliability of AI systems [68].

Validation and Generalizability

AI models often struggle with generalizability. Models developed in one clinical setting or population may not perform well in different environments, making it essential to validate AI systems across diverse patient groups and healthcare settings. This challenge underscores the importance of building robust, scalable AI tools that can deliver consistent performance across various clinical scenarios [69].

Patient Privacy and Data Security

The use of large datasets in AI brings critical concerns regarding patient privacy and data security. Compliance with regulations such as the Health Insurance Portability and Accountability Act (HIPAA) in the USA is vital to protect patient data. HIPAA ensures that individually identifiable health information is safeguarded and defines strict rules about how patient data can be shared, particularly when dealing with AI development that relies on large medical datasets [70].

To address these challenges, federated learning has emerged as a promising solution. Federated learning allows multiple healthcare institutions to collaborate on training AI models without directly sharing their sensitive data. This method enables AI algorithms to learn from decentralized data across multiple locations while maintaining data privacy. The system aggregates insights from local datasets into a global model, ensuring that no personal data leaves the individual institution, thus preserving patient privacy while still advancing AI's capabilities [71].

Bias and Fairness

AI algorithms can inadvertently perpetuate biases present in the data they are trained on, leading to disparities in healthcare outcomes. If the data used to train AI algorithms are not representative of diverse populations, AI systems could perpetuate or even exacerbate existing healthcare disparities. Ensuring that AI systems are trained on diverse and high-quality datasets is essential to minimize bias and ensure that AI-driven care is equitable across all patient demographics [72].

Regulation and Validation

For AI tools to be safely deployed in radiology, they must undergo rigorous validation and gain regulatory approval. This process can be lengthy and complex, in order to ensure the systems meet safety, accuracy, and efficacy standards. Regulatory agencies such as the Food and Drug Administration (FDA) ensure AI tools are compliant with clinical safety standards before they are used in patient care [73].

Impact on Radiologist's Role

The potential for AI to replace radiologists has been a source of concern; however, AI is more likely to augment the role of radiologists rather than replace them entirely. AI excels at identifying patterns and anomalies in medical imaging, but it lacks the clinical judgment and contextual understanding that radiologists provide. AI, while powerful in many aspects, cannot replicate the full depth of expertise required to interpret complex imaging findings. Furthermore, the "black box" nature of AI systems means that their decision-making processes are opaque and lack the transparency that radiologists provide when making clinical interpretations [74].

AI and radiologists work best in combination. For example, a radiologist-AI combination showed superior performance compared to either radiologists or AI alone in prostate cancer detection. This hybrid approach could potentially improve diagnostic accuracy, reduce physician workload, and enhance patient care quality [75].

AI serves as a valuable tool that enhances radiologists' efficiency, allowing them to focus on more complex cases while the AI handles routine tasks such as anomaly detection and data analysis. This symbiotic relationship between AI and radiologists can improve diagnostic workflows and ultimately improve patient care [76].

Evolution of AI technologies in radiology and future directions

As AI continues to evolve, its applications in radiology are expanding beyond current capabilities. With ongoing advancements, AI is poised to improve image quality, improve diagnostic accuracy, and provide personalized patient care.

Convolutional Neural Networks

A significant advancement in AI technologies within radiology has been the development of convolutional neural networks (CNNs). These deep learning models are designed to automatically analyze and interpret complex medical images, enhancing the accuracy of diagnostics. CNNs have become integral to AI applications in radiology, particularly in tasks such as detecting lesions, segmenting images, and classifying various conditions. Due to their ability to process large datasets with high precision, CNNs have contributed to improved diagnostic accuracy and reduced error rates. Their continued refinement is expected to further enhance radiological assessments and aid in early disease detection, making them a cornerstone of AI's future in radiology [9].

Radiomics and Precision Medicine

Radiomics is an emerging field that uses big data and AI to extract large numbers of quantitative features from medical images. These radiomic features, which are often beyond the capability of the human eye to detect, can reveal underlying tumor characteristics and patterns. By applying advanced data characterization algorithms, radiomics provides valuable insights into the tumor's heterogeneity, aiding in the diagnosis, prognosis, and treatment planning. This data-driven approach has the potential to significantly improve diagnostic accuracy and guide personalized treatment strategies.

In precision medicine, AI plays a critical role by identifying imaging biomarkers that correlate with specific clinical outcomes. These biomarkers can be used to predict how a tumor might respond to particular therapies, enabling clinicians to tailor treatments to individual patients. For example, AI can predict responses to chemotherapy or immunotherapy based on the tumor's radiomic profile, allowing for more targeted and effective interventions [77].

Furthermore, radiomics combined with machine learning models has shown promise in predicting disease progression, recurrence, and patient survival rates. As AI continues to advance, its integration with radiomics could facilitate even more personalized care by providing oncologists with precise data to inform treatment plans, particularly in cancer care. AI-powered radiomic analysis is likely to expand into other fields such as cardiovascular disease and neurodegenerative disorders, where subtle imaging changes can provide early indicators of disease [78].

Enhancing Computer-Aided Diagnostic Performance

Future AI systems are expected to integrate information from various imaging modalities such as CT, MRI, and PET to provide a more comprehensive analysis of a patient's condition. By combining multimodal imaging data with clinical data sources, AI systems will be able to offer insights that facilitate early diagnosis and personalized treatment plans. This integration of data holds great potential for improving outcomes in complex diseases such as cancer, where early and precise diagnosis is crucial [79,80].

Improving Image Quality

A key area where AI is making strides is in image quality enhancement. AI-powered algorithms can reduce noise and improve image resolution, which minimizes the need for repeat scans and helps reduce radiation exposure for patients. AI systems can also be used to adjust imaging protocols in real time, improving scan quality and shortening acquisition times, which further reduces patient discomfort and enhances productivity in radiology departments [9].

Applications of AI in radiology training and education

AI is increasingly influencing the way radiologists are trained, offering innovative methods for teaching complex cases and enhancing diagnostic skills. By simulating a wide array of clinical scenarios, AI-based platforms enable trainees to practice interpreting images from various modalities, such as X-ray, CT, and MRI, in a controlled environment. These systems can provide real-time feedback on performance, helping trainees refine their skills more effectively. For example, AI-assisted training tools can help identify missed diagnoses or incorrect interpretations, providing an opportunity for corrective learning and improvement [81,82].

AI can also be used to deliver "precision education" in radiology. By tailoring the learning experience to individual needs, AI systems can analyze each trainee's performance and adapt educational content accordingly, offering personalized guidance on areas that need improvement. This adaptive learning model is particularly beneficial for addressing the diverse learning styles and paces of different students. It has been shown that AI-driven adaptive learning platforms can improve the retention of knowledge and application in clinical practice [83].

Furthermore, AI-based educational tools help trainees by automating the analysis of large datasets, offering them hands-on experience with real-world radiological cases. These tools integrate with teaching platforms to present real clinical data, providing an experience that closely mimics daily radiology practice. When AI-assisted learning is incorporated into radiology residency programs, it can enhance the learning experience by making the process more interactive and data-driven [81].

## Conclusions

AI is revolutionizing the field of radiology, bringing significant advancements in diagnosis, workflow efficiency, and patient care. By automating routine tasks and providing more precise analysis, AI enables radiologists to focus on more complex cases, ultimately improving healthcare outcomes. AI-driven technologies, such as deep learning and machine learning, are enhancing image interpretation, streamlining workflows, and facilitating personalized treatment planning. However, the successful integration of AI into clinical practice requires overcoming several challenges. Ensuring data quality, maintaining algorithm transparency, and addressing privacy concerns are essential to building trust in AI systems. Moreover, continued collaboration between radiologists, AI developers, and regulatory bodies is critical to ensuring that these technologies are implemented safely and effectively.

Looking ahead, AI holds immense potential to further transform radiology, particularly in areas such as radiomics and precision medicine. As research and technological advancements continue, AI has the potential to play an even greater role in enhancing diagnosis and optimizing radiology workflows, ultimately improving patient outcomes.
